# Hepatitis C virus (HCV) infection among patients with sickle cell disease at the Korle-Bu teaching hospital

**DOI:** 10.1186/s12985-022-01797-z

**Published:** 2022-04-22

**Authors:** Gifty Mawuli, Bartholomew Dzudzor, Kenneth Tachi, Amma Anima Benneh-Akwasi Kuma, James Odame-Aboagye, Billal Musah Obeng, Anthony Twumasi Boateng, Elijah Paa Edu-Quansah, Keren Okyerebea Attiku, Esinam Agbosu, Augustina Arjarquah, Joseph Humphrey Kofi Bonney

**Affiliations:** 1 Department of Virology, College of Health Sciences, Noguchi Memorial Institute for Medical Research, University of Ghana, P. O. Box, LG 581, Legon Accra, Ghana; 2 Department of Medical Biochemistry, University of Ghana Medical School, University of Ghana, Accra, Ghana; 3 Department of Medicine and Therapeutics, University of Ghana Medical School, University of Ghana, Accra, Ghana; 4 Department of Hematology, College of Health Sciences, University of Ghana, Accra, Ghana; 5 Kirby Institute, University of New South Wales, Kensington, Australia; 6 African Field Epidemiology Network, Monrovia, Liberia

**Keywords:** Hepatitis C, Prevalence, Sickle cell anemia, Genotypes, HCV infection

## Abstract

**Background:**

Hepatitis C virus (HCV) infection is a blood borne infection that remains potentially transmissible through blood transfusions. Sickle cell disease (SCD) is a common inheritable haemoglobinopathy in Ghana that requires multiple blood transfusions as part of its management. The SCD patient is therefore at a high risk of HCV infection; however, data on the occurrence of HCV in SCD patients has not been documented in Ghana. This study sought to determine the prevalence and genotypes of HCV infection in SCD patients.

**Materials and methods:**

This was a cross-sectional study which enrolled 141 sickle-cell disease patients from the Ghana Institute for Clinical Genetics, Korle-Bu Teaching Hospital (KBTH). Patient information was obtained through a structured questionnaire. Aliquots of the plasma obtained was used for both serology with Advanced Quality Rapid Anti-HCV Test Strip and molecular testing by RT-PCR with primers targeting the HCV core gene. The amplified DNA were purified and subjected to phylogenetic analysis to characterize HCV genotypes.

**Results:**

Twelve (9%) out of the 141 patients were sero-positive for HCV total antibodies. HCV RNA was amplified from 8 (6%) out of the total number of patients’ samples. One of the 12 sero-positives was HCV RNA positive. Five (63%) out of the 8 HCV RNA positive samples were successfully sequenced. The phylogenetic tree constructed with the study and GenBank reference sequences, clustered all five study sequences into HCV genotype 1.

**Conclusion:**

The HCV seroprevalence of 9% among sickle cell disease patients is higher than reported for the general Ghanaian population which is 3%. Genotype 1 is the common HCV genotype infecting SCD patients. Sickle cell disease is likely to be a high-risk group for HCV inapparent infections in Ghana as seroprevalence does not correlate with viremia. However, even with higher seroprevalence, the group must be given priority in resource allocation for preventive, diagnostic and therapeutic strategies.

## Introduction

Globally, Sickle Cell Disease (SCD) is one of the most prevalent inheritable disorders mainly affecting people of African ancestry [1, 2]. Due to its common complication of chronic hemolysis or red cell breakdown, frequent blood transfusions have been part of the standard of care for SCD patients [3, 4].

Hepatitis C virus (HCV) infection is blood-borne with about 170 million chronically infected individuals worldwide [5]. Despite advances in HCV testing and transfusion medicine, it remains potentially blood transfusion transmissible infection-causing virus especially in sub-Saharan Africa due to uncertainties from testing and screening protocols. Prior to the availability of anti-HCV antibody testing at blood centers, post-transfusion HCV prevalence ranged from 2 to 30% [6]. However, current studies have shown that infections through blood transfusion have reduced significantly due to the better screening methods currently available [7].

The SCD patient remains at high risk of HCV infection because of their multiple blood transfusion requirements [8, 9]. Additionally, they experience more healthcare-setting contacts and hospitalizations than the non-SCD population. The combination of HCV and SCD results in devastating liver diseases including cirrhosis and hepatocellular carcinoma [10]. The presence of additional risk factors such as iron overload, immune suppression, and co-infection with other hepatotrophic viruses such as hepatitis B virus (HBV) help drive liver disease progression in SCD. The relationship between SCD and HCV therefore merits research attention [11].

There is a high burden of SCD in Ghana [12] and HCV is not uncommon with an estimated prevalence of 3.0% [13]. Majority of HCV carriers are unaware of the infection due its asymptomatic nature at the early stages [14, 15]. However, there is a dearth of information on the occurrence, treatment and genotypes of HCV among SCD patients in Ghana [14]. We sought to improve the knowledge gap by providing data on the occurrence and circulating genotypes of HCV among SCD patients at a tertiary health facility, Korle-Bu Teaching Hospital (KBTH) in Ghana.

## Materials and methods

### Study design and site

A cross-sectional, hospital-based study which involved 141 sickle cell disease patients was conducted at the Ghana Institute for Clinical Genetics (GICG) unit and Korle-Bu Teaching Hospital (KBTH) in the Greater Accra Region of Ghana. The KBTH serves as a tertiary referral health facility and is currently the third largest hospital in Africa. The GICG unit was established in 1974 and sees an average number of 40 patients daily with one third attending the center’s daycare services for management of crises.

### Data and sample collection

Demographic and clinical data including age, gender, nationality, origin, history of blood transfusion of consented and enrolled participants was obtained from the patients with a structured questionnaire. An average number of 10 participants were recruited in a day with one-time point sampling of clinical specimen of blood from consented participants by trained phlebotomists from the GICG unit. Three milliliters (3 ml) of venous blood were collected using a 5 ml syringe into an ethylenediamine tetra-acetic acid (EDTA) tube and labelled appropriately. The blood samples were transported in a cool box with ice packs to the Virology Department of the Noguchi Memorial Institute for Medical Research where the serological and molecular testing were done. Plasma was separated from the whole blood by spinning in a refrigerated centrifuge at 2500 rpm for 10 min, aliquoted and used for testing and with the residual specimen, cryopreserved at − 80 °C appropriately.

All serological and molecular tests for this study were done at the Virology Department in NMIMR which is recognized by the WHO and country -designated as a laboratory for testing emerging and dangerous viral pathogens of public health concern.

### Detection of anti-HCV antibodies

Plasma from all 141 blood samples were screened for anti-HCV antibodies using the Advanced Quality Rapid Anti-HCV Test Strip (InTec Products, INC, Xiamen, China). This kit uses the principle of immunochromatography and qualitatively detects antibodies to HCV in plasma, serum, and blood. The sensitivity of the kit is stated as 93% and specificity > 96.3% and is noted to be highest in serum and plasma versus whole blood. Fifty (50) µL of patient plasma was pipetted onto the sample pad of the test strip and two (2) drops of sample diluent were added to the sample pad after addition of the specimen. The results of the test were observed after 15 min. The test was said to be positive if both the control and test bands appeared.

### Genomic amplification and detection

Viral ribonucleic acid (RNA) was extracted using the QIAamp® RNA Viral Mini Kit (Qiagen, Hilden, Germany) following manufacturer’s instructions. Viral RNA was reverse transcribed at 50 °C for 30 min using the QIAGEN OneStep RT-PCR (QIAGEN, USA) and amplified by a conventional PCR with primers (sense (5′-CTT CAC GCA GAA AGC GTC TA-3′) and anti-sense (5′-CAA GCA CCC TAT CAG GCA GT-3′)) targeting HCV core gene. The PCR reaction volume of 25 µl constituted 5 µl of RNA extract, 5 µl of reaction Buffer (5X), 5 µl of nuclease free water, 1.5 µl (10 µM) of both forward and reverse primers to the 5′ untranslated region, 1 µl dNTPs (10 mM), 5 µl Q solution (5X) and 1 µl of enzyme mix [16]. Cycling condition was done at 95 °C for 15 min, 45 cycles of 95 °C for 15 s, 60 °C for 15 s and 72 °C for 30 s. One (1) µl of the amplicons from the first round PCR was used in a re-amplification protocol using the same set of primers at a total mix of 25 µl (16 µl of nuclease free water, 2.5 µl of Platinum buffer (10X), 1.5 µl of MgCl (50 mM), 0.5 µl of dNTPs (10 mM), 1.5 µl of both forward and reverse primers (10 µM) and 0.5 µl of platinum Taq) using the Platinum Taq DNA Polymerase kit (Invitrogen, Thermo Fisher Scientific). In this reaction, an initial denaturation of 95 °C for 3 min, 35 cycles of denaturation for 20 s at 95 °C, annealing at 55 °C for 20 s, extension for 60 s at 72 °C and extended finally for 20 min at 72 °C. The PCR products were visualized on a 2% agarose gel with gel red under ultraviolet trans-illumination using the BioDoc-It 220 Imaging System (Upland, CA, USA) to confirm an expected band size of 243 base pairs.

### Post PCR purification and cycle sequencing

The expected DNA fragments were cut out from the agarose gel with a clean, sharp surgical blade. This was done under a UV light. Purification of amplified PCR products was done using QIAquick PCR purification kit (QIAGEN, Hilden, Germany) following manufacturer’s protocol. Purified amplicons were eluted in 30 µl of elution buffer for cycle sequencing. Purified DNA was quantified using the Nanodrop 2000C spectrophotometer (Thermo Scientific, USA) prior to cycle sequencing. Cycle sequencing was done using the BigDye® Terminator v3.1 Cycle Sequencing kit (Applied Biosystems, CA, USA). A total reaction volume of 10 µl comprising of 2 µl (2 µM) each of a primer, BigDye terminator buffer, nuclease free water and purified PCR product were used. The cycling conditions were at 94 °C for 2 min, 25 cycles of 94 °C for 30 s, 50 °C for 15 s, 60 °C for 4 min and 4 °C hold. Sequenced products were purified using the Agencourt® CleanSEQ® Dye-Terminator Removal system (Agencourt Bioscience Corporation, U.S.A) following manufacturer’s protocol. The purified products were loaded onto the ABI 3130xl genetic analyzer (Applied Biosystems, MA, U.S.A) to generate sequence data.

### Nucleotide sequence analyses

Nucleotide sequences generated by the ABI 3130XL Genetic analyzer were viewed in Chromas software, (version 2.6.2) to detect background noise of the base calls. Sequences were then imported into the BioEdit version 7.2.5, trimmed, assembled to form a contig and manually edited (Fasta sequences available in S1-S5 Appendix). The reference strains for the different genotypes of HCV gene from the Genbank were compared to the consensus sequence obtained from the genetic analysis using the ClustalW multiple alignment accessory tool in BioEdit software version 7.2.5 [17] Phylogenetic analyses were done using the Molecular Evolutionary Genetics Analysis (MEGA X) software [18]. A bootstrap test and reconstruction were done 500 times to confirm the reliability of the phylogenetic tree.

### Statistical analysis

Data was entered into Microsoft Excel (2012) and analyzed using STATA version 13.1. The association between each variable (age, gender, blood transfusion, sickling genotype) and the dependent variable (anti HCV antibodies and HCV RNA) were tested. The Chi-square statistic test and the Fishers exact test where appropriate was used to determine the significance and strength of association at a confidence interval of 95% and a *p*-value of < 0.05.

## Results

### Demographic characteristics of study population

A total of 141 blood samples were collected from SCD patients who visited the day care clinic for their routine check-up at the Ghana Institute of Clinical Genetics–Korle-Bu. Study participants were patients diagnosed of any of the sickle cell disease types, SS, SC, SD, and SF (Table 1).

**Table 1:** Demographic distribution of SCD patients by age group, gender, sickling, and blood transfusion status at GICG, KBTH

Characteristics	Number of cases	Percentage
Overall total	141	100
*Gender*		
Males	51	36
Females	90	64
*Age group (years)*		
≤ 19	41	29
20–29	48	34
30–39	22	15
40–49	13	9
≥ 50	17	12
Median age	25 (12–79)	
*SCD type*		
SS	88	62
SC	47	33
SD	4	3
SF	2	1
*Blood transfusion*		
Yes	72	51
No	69	49

SS: Haemoglobin SS, SC: Haemoglobin SC, SD: Haemoglobin D-Punjab, SF: Fetal haemoglobin

Most of the study population were females representing 64% with the remaining 36% being males. Majority of the participants (35%) were within the age range of 20–29 years. The median age was 25 years (12–79 years). Majority (62%) of the study population were homozygous haemoglobin SS, with the lowest percentage (1%) been haemoglobin SF.

A little over half of the total population (51%) had received blood transfusion and the remaining 49% had never received blood transfusion (Table 1).

**Fig. 1:**
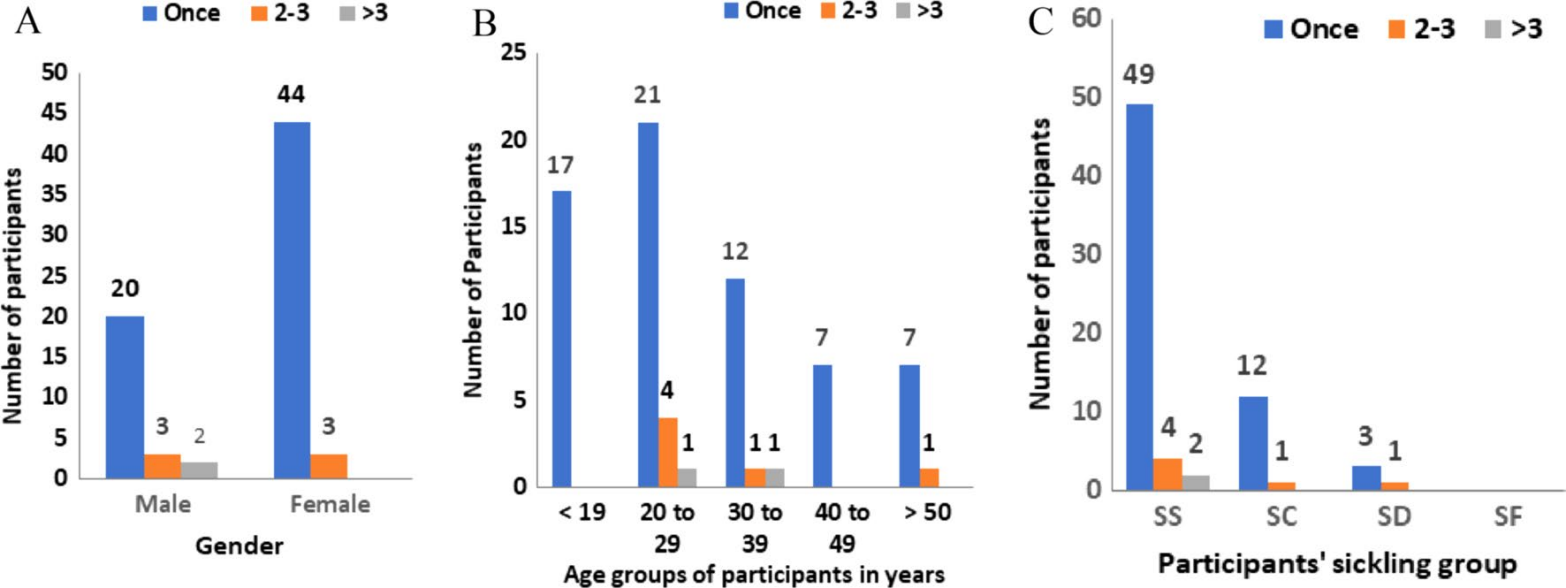
Distribution of the number of blood transfusion in SCD patients by: **A** Gender distribution: The number of times of blood transfusion in participants against sex. **B** Age group distribution: The number of times of blood transfusion in participants against age groups in years. **C** Sickling group: The number of times of blood transfusion in participants against sickling status

Of the 72 patients who had given blood, 64 (89%) had received it once, 6 (8%) have had transfusions twice and the remaining 2 (3%) had received transfusion more than 3 times (Fig. 1). None of the study participants knew their HCV status. The number of times a patient received blood transfusion as against gender, age groups and sickling status have been summarized in Fig. 1A–C.

### Detection of anti-HCV by rapid anti-HCV test

Our serological method established a 9% (12) prevalence of HCV antibodies among the 141 patients using the Advanced Quality Rapid Anti-HCV Test Strip (Table 2).

**Table 2:** Distribution of SCD patient by age group, sex, sickling genotype, and blood transfusion status against HCV detections at GICG, KBTH

Characteristics	No of patients N (%)	HCV (Serology)			HCV (RT-PCR)		
		Number of positives n (%)	Odds ratio (95% confidence interval)	*P*-value	Number of positives n (%)	Odds ratio (95% confidence interval)	*P*-value
Overall total	141 (100)	12 (9)			8 (6)		
*Gender*							
Males	51 (36)	8 (16)	4.00 (1.1504–14.1029) 1	0.0290^α^	3 (6)	1.0625 (0.2432–4.6418) 1	1
Females	90 (64)	4 (4)			5 (6)		
*Age group (years)*							
≤ 19	41 (29)	4 (10)	1.0811 (0.1819–6.4263)	0.9999	3 (7)	0.7895 (0.1218–5.1191)	0.9999
20–29	49 (35)	5 (10)	1.1628 (0.2075–6.5166)	1	1 (2)	0.4468 (0.0267–7.4900)	0.5329
30–39	22 (15)	2 (9)	1	1	1 (5)	1	
40–49	13 (9)	1 (8)	0.833 (0.0681–10.2021)	0.9999	1 (8)	0.833 (0.0681–10.2021)	0.9999
≥ 50	17 (12)	0 (0)	*	*	2 (12)	*	*
*SCD type*							
SS	88 (62)	7 (8)	0.8469 (0.2548–2.8153)	0.7664	5 (6)	1.0241 (0.2347–4.4685)	0.9999
SC	47 (33)	5 (11)	1.4966 (0.4485–4.9942)	0.5319	3 (6)	1.2273 (0.2805–5.3703)	0.9999
SD	4 (3)	0 (0)	*		0 (0)	*	
SF	2 (1)	0 (0)	*		0 (0)	*	
*Blood transfusion*							
Yes	72 (51)	4 (6)	0.4485 (0.1286–1.5640) 1	0.2377	2 (3)	0.3000 (0.0584–1.5646) 1	0.1599
No	69 (49)	8 (11)			6 (9)		

1 indicates reference variable

SS: Haemoglobin SS, SC: Haemoglobin SC, SD: Haemoglobin D-Punjab, SF: Fetal haemoglobin

*Calculation not applicable due to no positive detected

*p*-value set at < 0.05

^α^Significant finding

### Genomic detection of HCV RNA

**Fig. 2:**
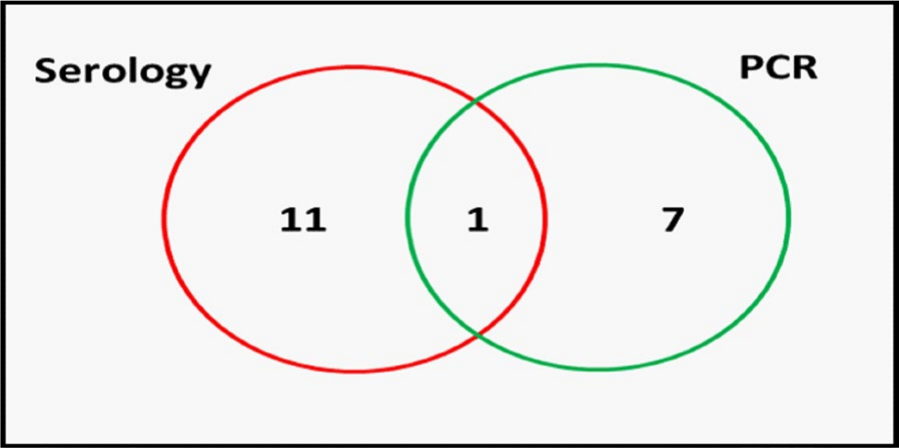
Relationship between the serologic and molecular testing. Red circle indicates the 12 patients who were HCV seropositive. The green circle indicates the total number of patients who were HCV RNA positive. One patient was positive by serology and PCR

The presence of the hepatitis C viral RNA was investigated in all 141 Sickle-cell disease patients by amplifying the core gene (C-gene) segment of the virus using polymerase chain reaction. A band size of 243 base pairs indicated successful amplification of the C-gene. Eight (8) of the 141 SCD patients had detectable viral RNA representing 6%. A representative gel is shown in Fig. 2.

### Comparison between serological and molecular HCV test results

Only one patient tested positive both for the Advanced Quality Rapid Anti-HCV Test Strip and PCR. The proportion of males’ positive (16%) for HCV antibodies was significantly higher than females (4%) with a *p*-value of 0.0281. The ratio of anti-HCV antibody positivity of males to females was about 4 times with an odd ratio of 4.04 (1.1540–14.1893).

None of the patients with SD and SF phenotypes patient tested positive for anti-HCV and HCV RNA. However, 8% and 6% positive cases of anti-HCV and HCV RNA respectively were found amongst patients with homozygous SS.

### Nucleotide sequence analyses

**Fig. 3:**
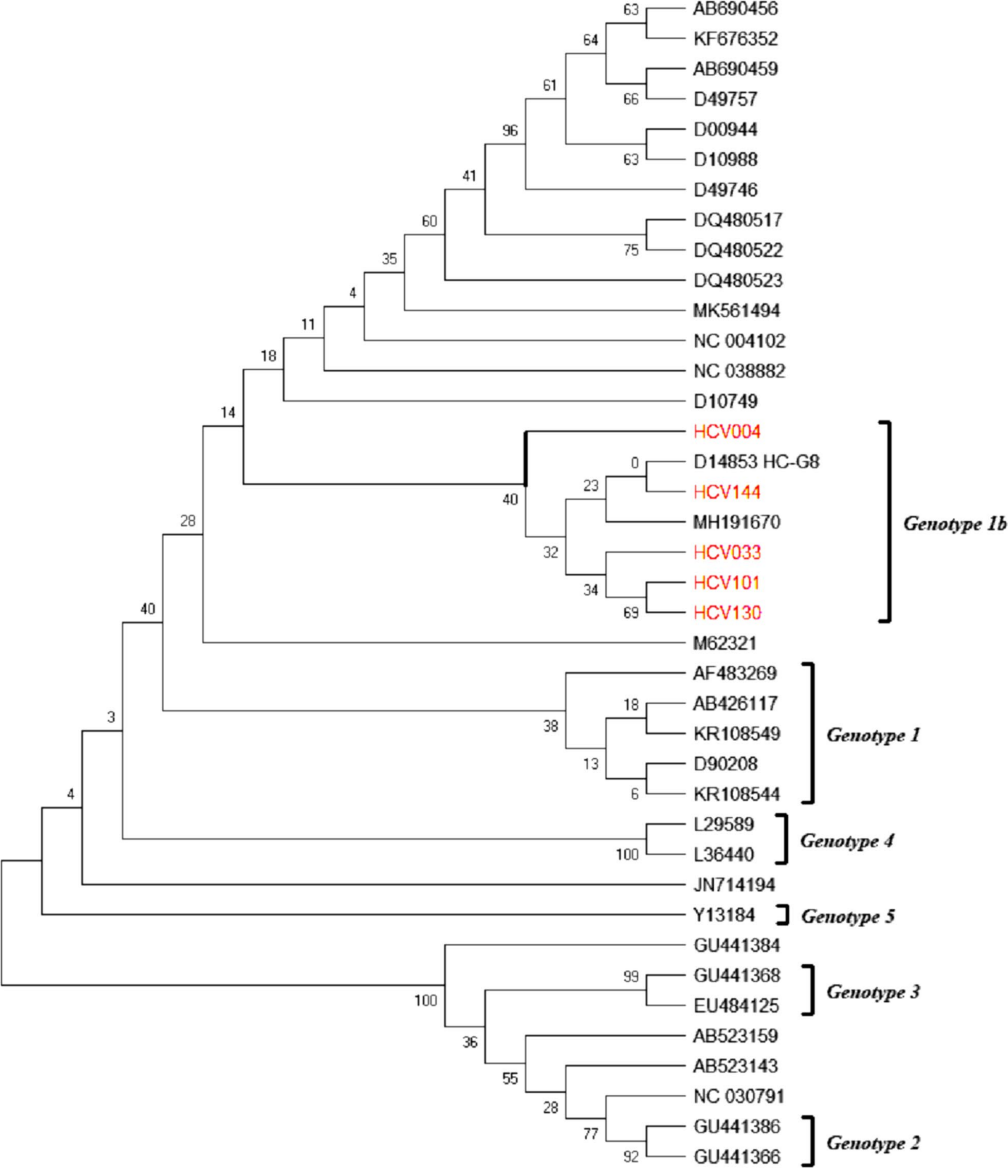
Phylogenetic relationship between HCV core sequences. Key: Samples from this study are in red font

Five (63%) of the 8 HCV-RNA positives were successfully sequenced for the core gene of hepatitis C virus. Thirty-eight (38) reference strains, representative for the different genotypes and sub-genotypes of HCV gene were obtained from the GenBank. Phylogenetic analysis was carried out to determine the relatedness of the identified genotypes in this study with reference strains. The Ghanaian HCV strains were closely related to each other and form within genotype 1, related to sub-genotype 1b. Genotype 1 isolates clustered with reference sequences, from different parts of the world (Fig. 3).

The evolutionary history was inferred by using the Maximum Likelihood method and Tamura-Nei model. The tree with the highest log likelihood (− 2174.67) is shown. The percentage of trees in which the associated taxa clustered together is shown next to the branches. Initial tree(s) for the heuristic search were obtained automatically by applying Neighbor-Join and BioNJ algorithms to a matrix of pairwise distances estimated using the Maximum Composite Likelihood (MCL) approach, and then selecting the topology with superior log likelihood value. This analysis involved 39 nucleotide sequences. There was a total of 247 positions in the final dataset. Evolutionary analyses were conducted in MEGA X.

## Discussion

This is the first documented report on the characteristics of HCV infection among SCD patients in Ghana according to our literature search. The sero-prevalence of HCV infection from this study is about 9%. This prevalence rate is comparatively higher than the national prevalence rate of 3% as reported in a review paper by Agyemang et al. [13]. The higher prevalence rate is comparable to similar studies done in Basra-Iraq and Washington DC, with anti-HCV sero-positivity rate among SCD patients being 9.2% and 10% respectively [11, 19]. The population variability and different testing algorithm could account for the differences in percentages [11, 20, 21]. In Ghana, an extremely high sero-prevalence of 11.6% and 19.2% was reported among jaundiced patients [22] and prisoners (higher risk of intravenous drug use) [23] respectively. This confirms a high sero-prevalence of HCV among selected population in Ghana, although the national prevalence is quite low.

This study also reports interesting findings on the utility of anti-HCV testing in SCD patients. One of the eight RNA positive samples also tested positive for anti-HCV, with the other seven been anti-HCV negative. A plausible explanation for this will be that 11 patients (Anti-HCV+, RNA−) had resolved HCV infection, one (anti-HCV+, RNA+) had chronic infection [14] and 7 (Anti-HCV−, RNA+) has probable acute infection. It is however also possible that the HCV test kit under-performed or the certain factors in the serum of the SCD patient militate against the sensitivity and specificity of the test in SCD patients. The implications of this finding if confirmed will be that anti-HCV Ab testing may not be a sufficient screening tool for HCV infection in SCD patients.

Our study recorded 11 (92%) out of the 12 anti-HCV antibodies positive patients who were HCV-RNA negative by PCR. We speculate that these 11 patients might have gone through a spontaneous viral clearance, which is undetectable HCV-RNA in patient’s plasma, but positive for anti-HCV antibodies in the absence of antiviral therapy [24]. In addition, plasma HCV RNA levels vary significantly overtime and this may result in a false-negative result mainly when the rate of viral replication is low. Another factor for a false negative result by PCR could be due to viral persistence limited to the liver. These factors could account for the low positivity rate of HCV-RNA. Results from this study is not different from similar studies conducted in sub-Saharan Africa which reported undetectable HCV-RNA between 41 and 89% of individuals who tested positive by serology [25, 26]. However, the different assays used makes the comparison difficult. Although this group have supposedly cleared the virus, anti-HCV antibodies usually persist for life and these neutralizing antibodies are not protective [27]. Reinfection with any genotype of HCV can occur in individuals who spontaneously cleared the virus [28].

As early as 7–21 days after HCV infection, the viral RNA can be detected by molecular testing [29]. A percentage of individuals who were exposed to HCV had detectable RNA but are anti-HCV antibodies negative [30]. This group of people are known to have late seroconversion which could last over a year [31]. This study recorded 7 out of 8 patients who had detectable HCV RNA by PCR but serologically negative. The core protein of HCV is said to appear in the early stage of the illness and since the PCR targeted the core region, we could suggest that these patients could have been in the early or acute phase of the infection.

Presumed chronic Hepatitis C infection with active virus replication was reported in a patient who tested positive by serology and PCR. Hepatitis C virus RNA detection by PCR confirms chronic HCV infection only when a serological test is reactive for anti-HCV antibodies [29, 32]. This patient had received transfusions in 2013, a year when screening for anti HCV RNA in blood donors was already in place. Torres et al. [33], who conducted similar research reported on an HCV chronically infected patient who had received blood transfusion during the screening era. It is difficult to ascertain whether the infection was through blood transfusion. Several studies have reported on the risk of HCV transmission through blood transfusion among SCD patients, however, we cannot conclusively associate HCV infection to blood transfusion. This is because a higher number of both HCV-Ab and HCV-RNA positives were recorded among study participants who had not been transfused (Table 2).

This study also confirms the male predominance of HCV antibody infection among SCD patients. This result is comparable to a study among SCD patients in Brazil which equally recorded predominance of HCV infection among males [33]. The male predominance is particularly worrying as other factors that accelerate HCV infection progression to cirrhosis such as frequent consumption of alcohol (> 2–3 drinks per day) and infection with other diseases such as hepatitis B and HIV [27, 34] are more predominant in males. Male SCD therefore should be a priority in decision making for HCV management. This is however interpreted with caution due to the small sample size for the study.

Infection with HCV has previously been shown to be directly linked to a history and number of blood transfusions. In this study however, no such correlation was established. More recent studies have also reported a reduction of HCV infection among blood transfused patients after the routine serological screening for anti HCV antibodies at blood banks was implemented [34, 35]. The current study may be a confirmation of the compliance to infection control protocol at the KBTH blood bank.

The prevalence of chronic HCV infections in sub-Saharan Africa ranges 0.7–1.6% [36]. In this region, the estimated sero-prevalence of HCV is 3.0% with an overall HCV RNA incidence of 0.7–1.6% [36]. Majority of patients with chronic HCV infection were not aware of their condition since HCV establishes infection by virtue of its long asymptomatic nature before seroconversion [14]. This creates a gap in testing leading to late diagnosis and hence hindering the elimination of HCV infection. Hepatitis C virus infection normally presents with no symptoms during the acute phase although 15–30% may experience non-specific symptoms [15, 37]. In the present study, none of the patients who tested positive either by serology or PCR was aware of their condition. This is no different from a similar study by Dustin et al. [15], who observed that patients who were HCV positives were unaware of the infection due to the absence of clinical symptoms at the early stages. There is therefore a need for a routine screening for HCV in high-risk populations.

The knowledge of HCV genotypes has an impact on the choice of therapy [14]. Although there are pan-genotypic therapies, these are yet to be registered in Ghana. This study showed clearly a 100% predominance of Genotype 1, specifically 1b in the study population. All the Ghanaian strains were closely related. This finding contradicts other studies conducted in other Ghana populations such as blood donors and or cohorts which reported genotype 2 as the highest circulating type in the country [20, 38]. Moreover, the different route of transmission has been associated with the circulating genotypes. Subtypes 1a, 1b, 2a, 2b, 2c and 3a have been related to parenteral routes [39]. We speculate that all the Ghanaian patients might have had the infection through a parenteral pathway. We, however, suggest additional larger studies on genotypic characterization to inform the circulating genotypes in the country.

The findings of this study are subject to several limitations. First, the sample size for was very low to draw inferences. Secondly, the study used only one antibody test which may have affected the sensitivity of antibody detection. Moreover, during the data capture, limited information was gathered about the clinical symptoms of the study participants hence our report on clinical manifestations of HCV in SCD was not adequate.

## Conclusion

Anti HCV seroprevalence among SCD patients at GICG in KBTH from our study is higher (9%) than reported for the general Ghanaian population (3%). Additionally, HCV RNA detection among HCV-Ab negative SCD patient is high. This suggests an inapparent infection amongst the study population. Our data further indicates that Genotype 1b is the common HCV genotype amongst infected SCD patients.

We recommend well-tailored preventive, diagnostic, and therapeutic interventions for better health outcomes. In the absence of pan-genotypic therapies, HCV genotype 1 therapies should also receive priority from drug importers and licensors.

## Data Availability

Data analyzed in this study were a re-analysis of existing data, which are openly available at locations cited in the reference section.

## Abbreviations

ABI: Applied Biosystems International
EDTA: Ethylenediamine tetra acetic acid
GICG: Ghana Institute for Clinical Genetics
HCV: Hepatitis C virus
KBTH: Korle Bu Teaching Hospital
MEGA: Molecular Evolutionary Genetics Analysis
SCD: Sickle cell disease
